# PsyCARE study: assessing impact, cost-effectiveness, and transdiagnostic factors of the Italian ministry of health’s “psychological bonus” policy

**DOI:** 10.1186/s40359-023-01345-6

**Published:** 2023-10-05

**Authors:** Ilaria M.A. Benzi, Angelo Compare, Antonino La Tona, Santo Di Nuovo, David Lazzari, Vittorio Lingiardi, Gianluca Lo Coco, Laura Parolin

**Affiliations:** 1 Department of Brain and Behavioral Sciences, University of Pavia, Pavia, Italy; 2 Department of Human and Social Sciences, University of Bergamo, Bergamo, Italy; 3 Department of Science of Education, University of Catania, Catania, Italy; 4 National Board of Italian Psychologists (CNOP), Rome, Italy; 5 Department of Dynamic and Clinical Psychology, and Health Studies, Faculty of Medicine and Psychology, Sapienza University of Rome, Rome, Italy; 6 Department of Psychology, University of Palermo, Palermo, Italy; 7 Department of Psychology, University of Milan-Bicocca, Piazza dell’Ateneo Nuovo, 1, Milan, 20126 Italy

**Keywords:** Psychological intervention, Mental health, Psychotherapy, PsyCARE, Transdiagnostic factors, Epistemic trust, Emotion regulation, Effectiveness, Anxiety, Depression

## Abstract

**Background:**

The prevalence of anxiety and depression disorders is surging worldwide, prompting a pressing demand for psychological interventions, especially in less severe cases. Responding to this need, the Italian government implemented the “Psychological Bonus” (PB) policy, allotting 25 million euros for mental health support. This policy entitles individuals to a minimum of four to twelve psychological sessions. In collaboration with the National Board of Italian Psychologists, our study assesses this policy’s effectiveness. Indeed, the PsyCARE study aims to examine the utilization of the Psychological Bonus, evaluate its impact on adult and adolescent participants’ psychological well-being through pre- and post-intervention assessments and six-month follow-up, and conduct a longitudinal cost-effectiveness analysis of this policy. A secondary aim is to investigate the influence of these interventions on transdiagnostic factors, including emotion regulation and epistemic trust.

**Methods:**

The study involves licensed psychotherapists and their patients, both adults and adolescents, benefiting from the Psychological Bonus. Data collection is underway and set to conclude in December 2023. Psychotherapists will provide diagnostic information and assess patient functioning. In addition, patients will be evaluated on mental health aspects such as clinical symptoms, emotion regulation, epistemic trust, and quality of life. We will employ linear mixed-effects models to analyze the outcomes, accounting for both fixed and random effects to capture the hierarchical structure of the data.

**Discussion:**

We anticipate the study’s findings will highlight reduced psychological distress and improved quality of life for participants and demonstrate the Psychological Bonus policy’s cost-effectiveness. The study will gather data on the role of specific versus nonspecific therapeutic factors in psychotherapy while adopting a patient-tailored approach to identify effective therapeutic elements and examine transdiagnostic factors. Overall, this study’s findings will guide future measures within the Italian healthcare system, fostering a psychological health culture and providing valuable insights to the broader public.

**Study registration:**

https://osf.io/6zk2j

## Introduction

### A global mental health emergency

A recent epidemiological study from 204 countries and territories highlighted approximately 53 million cases of major depressive disorder and 76 million cases of anxiety disorders directly linked to the pandemic. Notably, the younger population experienced the most significant increase, with women being twice as affected as men by these disorders [1].

In Italy, the Mental Health 2021 report published by the Italian Ministry of Health in 2022 stated that 778,737 individuals sought assistance from public specialist mental health services during 2021. Furthermore, nationwide data showed that 3.3% of emergency room admissions (n = 479,276) were related to psychiatric issues. Among individuals seeking assistance for mental health-related problems, 14.6% resulted in hospitalization, while 72.1% were managed at home. Thus, 7 out of 10 individuals seeking mental health services do not present with severe enough conditions to require hospitalization but require some form of psychological support, nonetheless. Moreover, 39.6% of all admissions are diagnosed with neurotic and somatoform syndromes [2]. Available data supports the evidence that the current public mental health system does not adequately address chronic cases, sub-clinical severity, or those that do not involve emergency management.

According to the 2022 Organisation for Economic Co-operation and Development (OECD) report titled “Health at a Glance: Europe,“ there was an increase in unmet needs for mental health care during and after the pandemic, with 23% of adults reporting such conditions in spring 2022, compared to 20% in spring 2021. While comprehensive data remains limited, national estimates indicate that the prevalence of depressive symptoms during the pandemic was approximately twice as high as pre-pandemic levels in numerous European countries [3]. Also, when looking at children and adolescents, recent estimates from the World Health Organization indicate that one in six individuals aged 10–19 have a mental disorder, with depression, anxiety, and behavioral disorders being the primary causes of distress [4]. Similar figures have been confirmed in Italian adolescents by a recent literature review by Deolmi & Pisani (2020) that emphasized the high prevalence of anxiety and depressive symptoms among young Italians attributed to the pandemic, social isolation, and parental stress [5].

In response to this situation, several European countries have implemented strategies to enhance mental health support, including establishing new information channels, expanding entitlements to mental health services, and increasing funding to improve the availability and accessibility of these services [3].

### The “Psychological Bonus”: a measure for the Italian population

Italy lacks a designated mental health budget for overall outpatient services. Recent evidence highlighted the benefits of measures such as an “Individual Health Budget” (resources allocated to meet specific health needs) to improve individuals’ mental health burden and promote a person-centered approach to mental health [6]. Nonetheless, available findings represent only a small population at a regional level.

However, in 2022, the Italian government introduced a new “Psychological Bonus” (PB) policy. This initiative allocated 10 million euros for mental health support, with an additional 15 million euros added in response to the numerous requests. The “Psychological Bonus” contributes to the costs of psychotherapy sessions to support people experiencing anxiety, depression, and psychological fragility due to the pandemic emergency, the consequent socio-economic crisis, and other psychological difficulties. Italian citizens residing in the country and with a yearly Equivalent Economic Situation Indicator (ISEE) equal to or below 50,000 Euros could apply for the funding [7].

The benefit is granted once for every applicant and must be used within 180 days after approval. If the application is approved, the contribution is awarded up to 50 Euros per psychotherapy session and distributed until the maximum allocated sum is reached. The given sum is up to 600 Euros for those with an ISEE lower than 15,000 Euros, up to 400 Euros for a beneficiary with an ISEE between 15,000 and 30,000, and up to 200 Euros for a beneficiary with an ISEE above 30,000 but not over 50,000 Euros.

Thus, the PB supports a minimum of four up to a maximum of twelve sessions, depending on the individual’s income. Furthermore, the benefit is available for new psychotherapies as well as for individuals that are already in treatment.

In December 2022, the number of funding requests reached 395,604. Nevertheless, funding constraints permitted the acceptance of only 10.52% of all applications, totaling 41,657. Once the applications were received, a ranking list was compiled, giving preference to individuals with lower incomes. Additionally, 27,280 psychotherapists willingly made their services available to the “Psychological Bonus” applicants.

To date, no data is available on its utilization and effectiveness.

Indeed, as anxiety and depression symptoms are prevalent in the general population, the PB is a first-time initiative. Thus it is crucial to explore to analyze the diagnostic characteristics of those who applied for this policy, both from the therapists’ and patients’ perspectives [8, 9].

### Efficacy and effectiveness of psychological interventions: a delicate balance

Evaluating the cost-effectiveness and tangible impact on individuals’ well-being is essential to justify government and institutions’ promotion and funding of psychological interventions.

*Efficacy* and *effectiveness* can be interpreted as extremes on a continuum in psychotherapy research [10]. Research on efficacy aims to evaluate a therapy under ideal and controlled conditions. This often includes randomized controlled trials (RCTs) carefully designed to minimize potential confounding variables. The study participants are systematically selected to meet specific inclusion and exclusion criteria, and the therapy is delivered in a standardized, controlled manner. Thus, the primary question in efficacy research is: “Does the therapy work in a controlled setting?“.

An exemplary case of research on *efficacy* is the Improving Access to Psychological Therapy (IAPT) project launched by the United Kingdom government in 2008 to address the need for psychological prevention and treatment measures [11]. With its focus on promoting mental health, IAPT offered evidence-based therapies for depression and anxiety disorders, including cognitive behavioral therapy and counseling, adhering to the National Institute for Health and Care Excellence (NICE) guidelines [12]. The IAPT model was built on three crucial features: a stepped care model, evidence-based and highly standardized treatments, and the consistent application of outcome monitoring. In addition, these services used a care model, which aimed to distribute scarce resources for psychological therapy by providing low- and high-intensity interventions, highlighting the importance of investigating the effectiveness of psychological interventions. As a result, people received support promptly, using the least intrusive intervention first [11, 13].

A decade later, there are more than 200 IAPT services throughout England, making it the world’s largest publicly funded and systematic implementation of evidence-based psychological care. Indeed, it was the world’s largest publicly funded psychological therapy initiative, as a meta-analysis conducted by Wakefield et al. (2021) showed, fostering a substantial decrease in depression and anxiety symptoms among patients post-treatment and a medium increase in their work and social adjustment [14]. The success of the IAPT model has led other countries, including Australia, Canada, Norway, and Japan, to develop similar systems [15–18]. The IAPT model holds promise for global adoption and evolution to meet populations’ diverse and expanding mental health needs worldwide, providing a virtuous example of how evidence-informed programs can reshape public psychological care [19].

An *efficacy* study, like the one promoted by the IAPT model, aims at assessing and providing the maximal therapeutic effect that treatment can achieve. However, when put into practice, limitations rooted in the complexities of the clinical practice itself might occur. For example, participants may not have the full range of comorbid conditions commonly seen in real-world settings, and the therapists might not be sufficiently trained or strictly supervised in their adherence to a model in most community settings [20–22].

To overcome these limitations, we can consider research on *effectiveness* on the other side of the continuum from research on *efficacy*. Indeed, *effectiveness* research aims to evaluate how therapy works in real-world conditions, measure the degree of its beneficial effects in “real-world” clinical settings, and consider the overall value of treatment as it would be applied in routine clinical practice. Participants in these studies represent a broader patient population, and the therapy is delivered more flexibly, which might better reflect typical clinical practice. *Effectiveness* research often considers a broader range of outcomes, including quality of life, function, and patient satisfaction, not just symptom reduction. Indeed, the primary question addressed in effectiveness research is: “Does the therapy work in everyday practice?“.

Indeed, literature has pointed out that, although a systematic approach allows for a clearer understanding of both the study of the process (“how does it work?“) and outcome (“does it work for…?“) of psychotherapy, also encountering the reality of clinical practice is crucial.

### Beyond Symptomatology: transdiagnostic factors

Recent literature has highlighted the utility of considering transdiagnostic factors in psychotherapy. Indeed, they refer to the underlying causes of mental health problems that might share commonalities amongst different symptomatic phenotypes and allow evaluation of the effectiveness of an intervention targeting factors such as emotion regulation and interpersonal functioning.

For example, emotion regulation is pivotal in mental health and well-being. A growing body of research suggests that deficits in emotion regulation are a critical transdiagnostic factor implicated in developing and maintaining various psychological disorders, notably anxiety and depression [23, 24]. Individuals with inadequate emotion regulation strategies often experience intense, prolonged negative affects and struggle to rebound from stressors, contributing to a heightened vulnerability to both anxiety and depressive symptoms. For instance, maladaptive emotion regulation strategies, such as emotional suppression, are frequently observed in these populations. These patterns of emotional dysregulation can result in a vicious cycle, where an inability to manage emotional responses effectively leads to increased psychological distress, further exacerbating difficulties with emotion regulation and perpetuating symptoms of anxiety and depression [25].

Emerging research suggests that deficits in epistemic trust (i.e., the capacity to trust the information others provide as reliable and personally relevant) may be another crucial transdiagnostic factor [26, 27]. Individuals with impaired epistemic trust often struggle with accepting and utilizing external guidance, hindering their ability to learn from therapeutic interventions, social feedback, or supportive interpersonal relationships. Furthermore, a decreased capacity for epistemic trust (i.e., epistemic mistrust) may also lead to an overreliance on internal, often negatively biased, interpretations of events and emotions, thereby perpetuating maladaptive cognitive processes and thought patterns [28–30]. As such, literature highlighted the importance of understanding the role of epistemic trust within the broader context of psychopathology and the therapeutic process.

## Aims and hypotheses

The “Assessing Impact, Cost-Effectiveness, and Transdiagnostic Factors of the Italian Ministry of Health’s “Psychological Bonus” Policy” study (PsyCARE) is anchored in this scenario.

Thus, the primary outcomes of the 36-month-long study entail: [1] exploring the access to the PB by analyzing the demographic characteristics of adhering therapists and users; [2] assessing the impact of interventions on the psychological well-being of users (adults and adolescents) investigating differences on indicators of psychological well-being between a baseline measurement (T0), a post-treatment measurement (T1) and a 6-months follow-up measurement (T2); [3] assessing the economical impact of the PB conducting a cost-effectiveness analysis that considers the benefits in terms of improved psychological well-being of users between a baseline measurement (T0) and a post-treatment measurement (T1), considering both direct health care costs (cost of treatment) and indirect costs (loss of user productivity).

Finally, a secondary outcome of the study is exploring the impact of psychological interventions on emotion regulation and epistemic trust [23, 24, 26, 27].

Thus, four main hypotheses will be tested in line with the available literature.

First, both the therapists participating in the study, beyond their theoretical frameworks, and patients will report mainly anxious and depressive symptoms [8, 9].

Second, the longitudinal trajectories for all psychological well-being indicators will improve between a baseline measurement (T0), a post-treatment measurement (T1), and a follow-up measurement (T2). More specifically, we expect psychological distress, anxiety, and depression symptoms to improve after treatment for adult and adolescent patients and to remain stable at a six-month follow-up [14].

Third, the PB will be a cost-effective initiative in terms of improved psychological well-being of users (adults and adolescents) between a baseline measurement (T0) and a post-treatment measurement (T1), accounting for direct healthcare costs (cost of treatment) and indirect costs (loss of user productivity). Thus, we expect the intervention will improve participants’ quality of life and productivity (i.e., loss of days of school/work because of ill health).

Fourth, emotion regulation, epistemic mistrust, and epistemic credulity will improve between a baseline measurement (T0), a post-treatment measurement (T1), and a follow-up measurement (T2). Moreover, we expect emotion regulation and epistemic trust to impact the longitudinal trajectories of psychological symptoms.

## Methods

### Study design

The PsyCARE study includes observational studies that are both cross-sectional and longitudinal. The University of Milan-Bicocca coordinates the study in collaboration with the University of Bergamo, the University of Catania, the University of Palermo, the University “La Sapienza” of Rome, and the National Board of Italian Psychologists.

The research study strictly adheres to the ethical guidelines outlined by the American Psychological Association (APA) and the principles outlined in the Declaration of Helsinki (seventh revision, 2013). The Ethical Committee of the University of Milan-Bicocca approved all materials and procedures.

### Sampling plan

Data collection will include two samples. The first sample will consist of therapists registered with the National Board of Italian Psychologists, adhering to the Italian Psychologist Bonus initiative. The second sample will consist of participating therapists’ patients encompassing adults over 18 years old and adolescents aged between 14 and 18 using the Psychologist Bonus.

Therapists are recruited via online newsletters and web news promoted by the National Board of Italian Psychologists. Patients are recruited via their therapists’ participating in the study.

Before participating in the study, all participants will provide informed consent. For under-age participants, informed consent will be obtained from adolescents and their parents/legal guardians. Participation is entirely voluntary. As an incentive for enrollment, therapists can participate in a training course on psychological assessment for free and obtain forty out of the fifty yearly professional mandatory training credits [31].

Each therapist is assigned a unique six-letter reference code (i.e., ABCDEF). To ensure patients’ matching, the therapist will assign their unique code and a number for every patient enrolled (i.e., ABCDEF-1 for patient number 1, ABCDEF-2 for patient number 2, etc.). All self-report questionnaires are completed via a secure web link to ensure anonymity on the Qualtrics platform.

Our target sample size, is a minimum of 450 therapists and 450 patients (adults and adolescents) to achieve sufficient power for the study.

### Data collection and measures

Data collection will include three-time points: at the beginning of the sessions funded by the Psychological Bonus (T0), at the end of the sessions funded by the Psychological Bonus (T1), and at six months follow-up (T2).

**Fig. 1:**
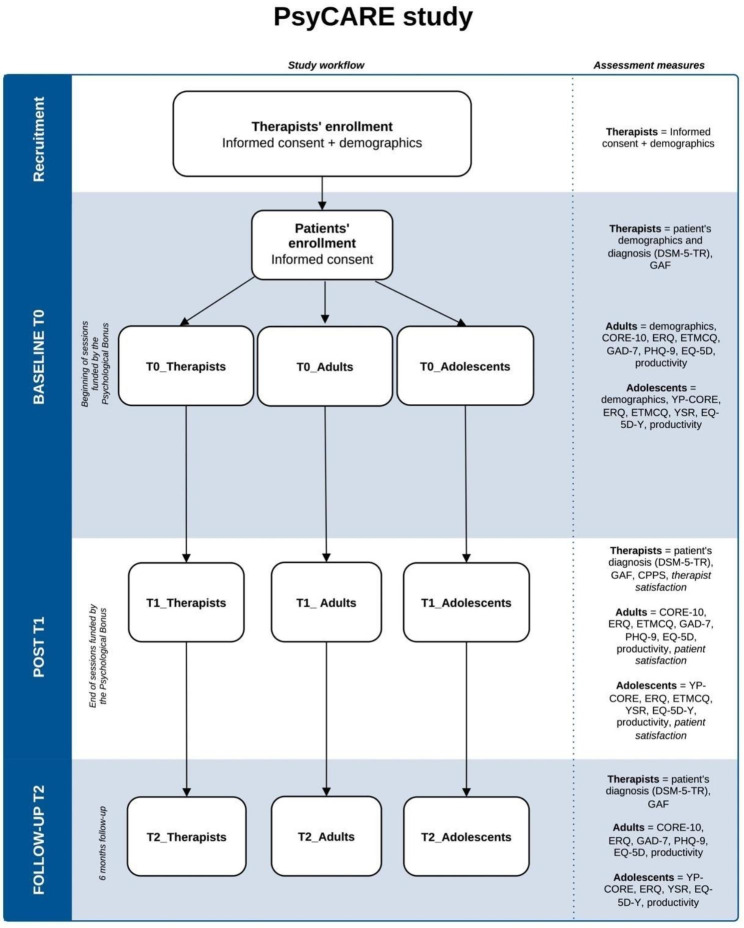
PsyCARE study flowchart. Note: **DSM**: Diagnostic and Statistical Manual of Mental Disorders Text Revision (APA, 2022); **GAF**: Global Assessment Functioning (GAF; Moos et al., 2002; Pedersen & Karterud, 2012); **CORE-10**: Clinical Outcomes in Routine Evaluation 10 (Barkham et al., 2013; La Tona et al., 2023); **YP-CORE**: Young Persons’ Clinical Outcomes in Routine Evaluation 10 (Twigg et al., 2016; Di Biase et al., 2021); **ERQ**: Emotion Regulation Questionnaire (Gross and John, 2003; Balzarotti et al., 2010); **ETMCQ**: Epistemic Trust, Mistrust and Credulity Questionnaire (Campbell et al., 2021; Liotti et al., 2023); **GAD-7**: Generalized Anxiety Disorder Scale (Spitzer et al., 2006); **PHQ-9**: Patient Health Questionnaire-9 (Kroenke et al., 2001); **EQ-5D**: Euro Quality Of Life 5D (Stolk et al., 2010; Balestroni & Bertolotti, 2012); **EQ-5D-Y**: Euro Quality Of Life Youth 5D (Wille et al., 2010; Scalone et al., 2011); **YSR**: Youth Self-Report (Achenbach, 1991); **CPPS**: Comparative Psychotherapy Process Scale (Hilsenroth, et al., 2005; Gentile et al., 2020)

Figure 1 illustrates the data collection process and measured variables.

Variables explored will vary according to the data collection phase.

### Therapists’ recruitment

After providing informed consent, therapists will provide demographic data such as gender, age, and information on years of professional experience, primary theoretical orientation, and primary activity settings (i.e., in person, online, or both).

### Patients’ recruitment and baseline assessment (T0)

#### Therapists assessment at T0

After an initial consultation with new patients or when starting the sessions funded by the PB for patients already in treatment, therapists will provide a baseline assessment of their patients providing demographic data, number of sessions already conducted, reason for contact, clinical history (i.e., previous psychological support), and diagnostic information [32].

The *Global Assessment of Functioning Scale* (GAF) [33–35] will also assess the patient’s overall mental well-being, from severe mental health disorders to optimal mental health. The GAF score falls between 1, indicating the most extreme illness, and 100, indicating optimal mental health. The scale is split into ten segments, starting from 1 to 10 and ending at 91 to 100. A score of 70 or above identifies minimal impairment, while scores below 60 indicate severe impairment.

#### Adult patients assessment at T0

After providing informed consent, patients aged > 18 will provide demographics, information on productivity (i.e., how many days of work/university/school did you lose because of your psychological problems?), and self-report on psychological distress, emotion regulation, anxiety and depression symptoms, epistemic trust, and quality of life.

The *Clinical Outcomes in Routine Evaluation-10* (CORE-10) [36] is a brief 10-item measure to explore psychological distress developed for routine use in practice settings. The CORE-10 is a shortened version of the 34-item CORE-OM [37]. It taps into three domains: i) problems: depression (2 items), anxiety (2 items), physical (1 item), and trauma (1 item); ii) functioning: general functioning (1 item), social functioning (1 item), and close relationships (1 item); and iii) risk: to self (1 item). In addition, two items (i.e., item 2, ‘I have felt I have someone to turn to for support when needed’ and item 3, ‘I have felt able to cope when things go wrong’) are worded positively and thus are reverse scored. Items are rated on a 5-point Likert-type scale (0 = not at all to 4 = most or all of the time), and higher total scores (i.e., the sum of all items, ranging from 0 to 40) indicate greater distress. The CORE-10 has been validated on the Italian population [38].

The *Emotion Regulation Questionnaire* (ERQ) [39] is a ten-item questionnaire measuring two emotion regulation strategies. Cognitive Reappraisal (six items) is a cognitive strategy consisting of the attempt to change the emotional impact of a situation by reinterpreting its meaning (e.g., “When I want to feel less negative emotion, I change the way I am thinking about the situation”). By contrast, Emotional Suppression (four items) directly targets expressive behavior as it consists of the attempt to inhibit the overt expression of emotions (e.g., “I control my emotions by not expressing them”). Instructions ask the respondent “some questions about your emotional life, in particular, how you control (that is, regulate and manage) your emotions.“ The ten items are rated on a Likert scale ranging from one (strongly disagree) to seven (strongly agree). For our study we will use the Italian adaptation of the ERQ as a reference [40].

The *Generalized Anxiety Disorder Scale-7* (GAD-7) [41, 42] is a 7-item, 4-point rating scale developed to assess how frequently the patient has experienced seven anxiety symptoms during the last two weeks: [1] feeling nervous or anxious; [2] being able to stop or control worrying; [3] worrying too much about different things; [4] difficulty relaxing; [5] being restless; [6] feeling easily annoyed or irritable; and [7] feeling frightened as if something terrible might happen. The response options are not at all (score = 0), several days (score = 1), more than half of the days (score = 2), and nearly every day (score = 3). In addition, an item is included to assess the duration of anxiety symptoms. The total GAD-7 score ranges from 0 to 21, with higher values indicating more anxiety symptoms. For this study, we used the Italian version of the GAD-7, which was recently tested during the COVID-19 pandemic [43].

The *Patient Health Questionnaire-9* (PHQ-9) [44] is a 9-item, 4-point rating scale developed to assess depressive symptoms the patient has experienced in the last two weeks. The response options are not at all (score = 0), several days (score = 1), more than half of the days (score = 2), and nearly every day (score = 3). The total PHQ-9 score ranges from 0 to 27, with higher scores indicating more severe symptoms. The PHQ-9 validity has been confirmed on the Italian population [43, 45].

The *Epistemic Trust, mistrust, and Credulity Questionnaire* (ETMCQ) [28] is a 15-item self-report questionnaire assessing Epistemic Trust, Mistrust, and Credulity toward communication or communicated knowledge. Epistemic trust refers to an adaptive attitude in relatively benign social circumstances in which the individual is selectively and appropriately open to social learning opportunities in relationships. Epistemic Mistrust reflects the tendency to treat any source of information as unreliable or ill-intentioned, trying to avoid being influenced by the communication of others. Epistemic credulity refers to a marked lack of vigilance and discrimination, signaling a general lack of clarity about one’s position and resulting in vulnerability to misinformation and the potential risk of exploitation. Higher scores indicate a higher presence of the relative trait for each factor. For our study we will use the Italian adaptation of the ETMCQ as a reference [46].

*The Euro Quality Of Life-5D* (EQ-5D) [47] is an instrument that evaluates the quality of life. The EQ-5D descriptive system is a preference-based health-related quality-of-life measure with one question for each of the five dimensions: mobility, self-care, usual activities, pain/discomfort, and anxiety/depression. The answers given to EQ-5D yield 243 unique health states or can be converted into an EQ-5D index, a utility score anchored at zero for death and one for perfect health. The questionnaire includes a Visual Analog Scale (VAS) by which respondents can report their perceived health status with a grade ranging from 0 (the worst possible health status) to 100 (the best possible health status). The EQ-5D has been validated on the Italian population [48].

#### Adolescent patients assessment at T0

After collecting informed consent from both adolescents and their parents, patients aged between 14 and 18 will provide demographics, information on productivity (i.e., how many days at school did you lose because of your psychological problems?), and self-report on psychological distress behavioral problems, emotion regulation (ERQ), epistemic trust (ETMCQ), and quality of life.

The *Young Persons’ Clinical Outcomes in Routine Evaluation* (YP-CORE) [49] is a brief 10-item measure to explore psychological distress in adolescents. Items are inspired by the CORE-OM and explore general well-being, symptoms/problems, functioning, and risk for the self. The questions all focus on how the person has felt in the past week. Each question has five possible answers, ranging from 0 = “Not at all” to 4 = “Most or all of the time”. The total clinical score is the sum of all scores (range 0–4) ranging from zero to 40. For our study, we wil reference the Italian validation of the YP-CORE [50].

The *Youth Self Report* (YSR) [51] is a 112-item self-report measure that assesses general psychological and behavioral difficulties. The YSR is the most widely used scale for assessing behavioral problems in adolescence, supported by excellent psychometric properties [52] Each item is scored on a 3-point scale (0= “not true” to 2= “very or often true”). The measure yields a Total Problems score of general pathological functioning and two comprehensive subscales of Externalizing behavior problems and Internalizing problems. The Externalizing scale encompasses the subscales of Aggressive behaviors and Rule-breaking behaviors. The Internalizing scale includes the Anxious/Depressed, Withdrawn/Depressed, and Somatic Complaints subscales. For this study, we will utilize the Externalizing and Internalizing problems scales and the Thoughts problems scale (e.g., strange behaviors, hallucinatory experiences, sleeping less). Higher scores indicate higher problems in the specific dimension.

*The Euro Quality Of Life-5D-Youth* (EQ-5D-Y) [47] encompasses five items exploring adolescents’ quality of life exploring their perceived level of problems (no difficulty, some difficulty, a lot of difficulty) in mobility, self-care, usual activities, pain or discomfort and anxiety or depression. The questionnaire includes a Visual Analog Scale (VAS) by which respondents can report their perceived health status with a grade ranging from 0 (the worst possible health status) to 100 (the best possible health status). The EQ-5D-Y has been validated on the Italian population [53].

### Post-intervention assessment (T1)

#### Therapists assessment at T1

At the end of the sessions funded by the PB, therapists will provide a post-intervention assessment of their patients providing diagnostic information [54], an evaluation of the patient’s overall mental well-being (GAF), information on their satisfaction level regarding the Bonus measure and on more technical aspects of their intervention.

The *Comparative Psychotherapy Process Scale* (CPPS) [55] is a 20-item self-report measure that can be used by clinicians, patients, or external observers, to assess the therapist’s techniques during a session highlighting psychodynamic-interpersonal techniques (PI; include psychodynamic, psychodynamic-interpersonal, and interpersonal therapy) and cognitive-behavioral techniques (CB; include cognitive, cognitive-behavioral, and behavioral treatment) on a 7-points Likert scale (0 = “Not characteristic” to 6 = “Extremely characteristic”). For our study, we will reference the Italian validation of the CPPS [56].

#### Adult patients assessment at T1

At the end of the sessions funded by the PB, patients aged > 18 will provide information on productivity (i.e., how many days of work did you lose because of your psychological problems?) and self-report on psychological distress (CORE-10), emotion regulation (ERQ), anxiety (GAD-7) and depression symptoms (PHQ-9), epistemic trust (ETMCQ), quality of life (EQ-5D), and level of satisfaction regarding the Bonus measure.

#### Adolescent patients assessment at T1

At the end of the sessions funded by the PB, patients aged between 14 and 18 will provide information on productivity (i.e., how many days at school did you lose because of your psychological problems?) and self-report on psychological distress (YP-CORE), behavioral problems (YSR), emotion regulation (ERQ), epistemic trust (ETMCQ), quality of life (EQ-5D-Y), and level of satisfaction regarding the Bonus measure.

### Follow-up assessment after six months (T2)

Six months after the end of the sessions funded by the PB, therapists will provide information on the continuation vs. interruption of their psychological interventions and eventually report on their patients’ diagnosis (APA, 2022) and overall mental well-being (GAF).

Adult patients will report on productivity, psychological distress (CORE-10), emotion regulation (ERQ), anxiety (GAD-7) and depression symptoms (PHQ-9), epistemic trust (ETMCQ), and quality of life (EQ-5D). Adolescent patients will report on productivity, psychological distress (YP-CORE), behavioral problems (YSR), emotion regulation (ERQ), epistemic trust (ETMCQ), and quality of life (EQ-5D-Y).

## Data analyses

​​Statistical analyses will be conducted using R Core Team ver. 4.3.1 [57].

We will compute descriptive statistics for all study objectives, utilizing the *psych* package [58], to examine the participants’ general characteristics.

We will compute descriptive statistics to explore access to the PB by analyzing the demographic characteristics of adhering therapists and patients (Objective 1). This will allow us to examine the characteristics of participants in terms of age, gender, socioeconomic status, and geographical location, among other pertinent variables (i.e., therapists’ expertise and theoretical framework; patients’ diagnostic information and severity of psychological distress).

To assess the impact of interventions on the psychological well-being of users (Objective 2), we will employ mixed models with random coefficients across participants using the R package *lme4* [59].

We conducted a power analysis using a simulation-based approach to estimate the minimum sample size required to detect a small effect size. The simulations were conducted using the R package *simr* [60]. The data simulation model was configured with random intercepts for participants. We assumed that all random variances were equal to 1, and the effect size of the outcome (slope) was computed to correspond to a Cohen’s d of 0.20 [61]. The correct slope coefficient value was calculated using the formulas described in Judd, Westfall, and Kenny [62]. Setting the simulations model with coefficients for the target effect random across participants ensures a pessimistic, and therefore more conservative, estimation of the minimum number of participants required [63]. According to the design, we varied the number of participants until the simulations showed the required power (1 − β = 0.80). The results of the power analysis indicated that a sample of N = 220 participants would provide a power of 0.80, and a sample of N = 290 would yield a power of 0.90. Accounting for a 50% dropout rate, we will to collect a minimum of 435 participants.

To assess the economic impact of the PB (Objective 3), we will conduct a cost-effectiveness analysis (CEA) [64]. The calculation will include both direct and indirect costs between the baseline (T0) and post-intervention (T1) assessments. Direct healthcare costs will encompass the costs of the psychological intervention and any related healthcare costs. These costs will be compared to data on standard care from the Italian Ministry of Health to calculate the incremental costs of the intervention. Indirect costs, or productivity losses, will be estimated by multiplying the number of days of work/university/school lost due to psychological problems (as reported by participants) by an average daily wage (for work) or an estimated cost of a lost day of education (for university/school). The effectiveness of the intervention will be measured using the EQ-5D, which will be used to estimate Quality Adjusted Life Years (QALYs). The incremental cost-effectiveness ratio (ICER) will be calculated as the cost difference between the PB intervention and standard care from T0 to T1, divided by the difference in QALYs over the same period. The resulting ICER will represent the additional cost per QALY gained by the PB intervention compared to standard care. A sensitivity analysis will be conducted to test the robustness of our findings to changes in key assumptions or parameters, including the unit costs used to calculate direct costs and the valuation of lost productivity. This analysis will be conducted in accordance with standard guidelines for conducting and reporting CEAs, such as the Consolidated Health Economic Evaluation Reporting Standards (CHEERS) statement [65]. The power analysis from the *pwr* package in R [66] indicated that for a paired t-test with a small effect size (Cohen’s d = 0.20), a power of 0.80, and a significance level α = 0.05, you would need a sample size of approximately n = 199 pairs of observations.

To assess the hypotheses on emotion regulation and epistemic trust (Objective 4), we use mixed models with random coefficients across participants. We conducted a power analysis using a simulation-based approach to estimate the minimum sample size required to detect a small effect size using the R package ‘simr’ [60]. We included the interaction of time with the transdiagnostic factor (i.e., emotion regulation) and their main effects. The results of the power analysis indicated that a sample of N = 200 participants would provide a power of 0.80, and a sample of N = 280 would yield a power of 0.90. Accounting for a 50% dropout rate, we will to collect a minimum of 420 participants.

The number of sessions conducted will be included as a covariate in all our multilevel models to control for potential confounding effects. This allows us to account for variability in therapy length, as the number of sessions may range from 4 to 10 per the parameters of the PB, but may extend beyond that if patients continue the intervention. Moreover, to account for differences between individuals that where already in treatment and not, we will include group variability in our models.

We will conduct post-hoc tests and sensitivity analyses to explore all findings further.

## Discussion

The PsyCARE study’s primary goal is to provide systematic data on the Italian Government’s “Psychological Bonus” effectiveness in promoting access to psychological treatments. The study breaks ground in several ways.

First, the PB represents a first-time national initiative due to its promotion by the Ministry of Health outside the public healthcare system. However, funding is limited as only 10.52% of applicants will be allowed to benefit from professional interventions for psychological problems. The situation of public services in Italy has shown their struggle in responding to the great demand for intervention for psychological problems that do not require urgent treatment. This insufficient response risks contributing to the chronicization or worsening over time of situations that, if intercepted earlier, might instead have a different prognosis. Thus, the PsyCARE study will help provide more information on the effectiveness of the psychological interventions funded by the PB, both in lowering patients’ psychological distress and providing a cost-effective measure. Thus, the study’s data may help justify future financial allocations and influence policymakers to strengthen public and private services.

Secondly, PsyCARE will gather real-world data on the effectiveness of psychological interventions in reducing symptoms, with a particular focus on anxiety and depression. Indeed, the study seeks to integrate cross-sectional perspectives and evaluate the effects of short-term psychological interventions. The study’s results will potentially significantly contribute to the ongoing debate in psychotherapy regarding the role and impact of specific versus nonspecific therapeutic factors.

Third, in line with recent advancements in psychotherapy research, we aim to adopt a patient-specific, or “tailored,“ approach, exploring “what works for whom,“ as described by Peter Fonagy: indeed, the PsyCARE will provide findings to identify the therapeutic elements associated with patients’ improvement and determine which patients may benefit the most from the bonus sessions and specific psychotherapeutic techniques adopted [67, 68]. For example, we will consider between-subjects variability on the longitudinal trajectories of psychological symptoms accounting for different diagnostic presentations and controlling for the therapists’ different theoretical frameworks. Moreover, the study will allow the evaluation of the effectiveness of a psychological intervention through the observation of transdiagnostic factors (i.e., emotion regulation, epistemic trust) to open a window of understanding of the mechanisms of change that can be influenced by psychological interventions and can, in turn, contribute to the reduction of symptomatic manifestations.

Fourth, the PsyCARE study aligns with the need to make scientific results replicable. Indeed, one of the major limitations of research in psychology is the inability to replicate the results of studies on different samples. Although the study guarantees a sufficient number for the reliability of its results, it will be necessary to replicate its findings in other contexts and populations. Thus, all data and materials will be available on the Open Science Framework [69].

Finally, the limitations of the study will be acknowledged in the study implementation and in interpreting its results. For example, the evaluation of patients may be time-consuming. However, collaboration and engagement of therapists in the study may decrease the rejection rate from patients. In addition, the evaluation will be done by administering self-report instruments, which could lead to bias in interpreting the results. However, collecting the therapist’s perspective might also help account for possible bias. Moreover, as PsyCARE includes longitudinal data collection, we might expect participants to withdraw or drop out of the study. Thus, statistical analysis will have to account for attrition to assess the robustness of the findings (i.e., sensitivity analyses). Finally, as both new patients and patients already in treatment could benefit from the PB, the number of sessions might represent a confounding variable in evaluating the psychological interventions’ efficacy. Thus, data analyses will account for these differences and explore any variability in longitudinal trajectories of relevant variables between patients already in treatment and new ones.

## Data Availability

Datasets generated and analyzed during the current study and statistical materials will be available on the Open Science Framework (OSF) project page (https://osf.io/u3apv/). The study’s pre-registration is available on the OSF [70].

## Declarations

### Study status

The current protocol is updated to June 2023. Data collection started on January 2023. The study is expected to end in December 2024.

## List of abbreviations

ISEE: Equivalent Economic Situation Indicator
RCT: Randomized Controlled Trial
IAPT: Improving Access to Psychological Therapy
CBT: Cognitive Behavioral Therapy
NICE: National Institute for Health and Care Excellence
GAF: Global Assessment of Functioning
CORE-10: Clinical Outcomes in Routine Evaluation-10
ERQ: Emotion Regulation Questionnaire
GAD-7: General Anxiety Disorder-7
PHQ-9: Patient Health Questionnaire
ETMCQ: Epistemic Trust, Mistrust, and Credulity Questionnaire
EQ-5D: Euro Quality of Life-5D
YP-CORE: Young Persons’ Clinical Outcomes in Routine Evaluation
YSR: Youth Self Report
EQ-5D-Y: Euro Quality of Life-5D-Youth
CPPS: Comparative Psychotherapy Process Scale
CEA: Cost-Effectiveness Analysis
QALYs: Quality Adjusted Life Years
CHEERS: Consolidated Health Economic Evaluation Reporting Standards
